# Shortened duration and reduced area of frozen soil in the Northern Hemisphere

**DOI:** 10.1016/j.xinn.2021.100146

**Published:** 2021-07-21

**Authors:** Ting Li, Yong-Zhe Chen, Li-Jian Han, Lin-Hai Cheng, Yi-He Lv, Bo-Jie Fu, Xiao-Ming Feng, Xing Wu

**Affiliations:** 1 State Key Laboratory of Urban and Regional Ecology, Research Center for Eco-Environmental Sciences, Chinese Academy of Sciences, Beijing 100085, China; 2 University of Chinese Academy of Sciences, Beijing 100049, China; 3 State Key Laboratory of Earth Surface Processes and Resource Ecology, Faculty of Geographical Science, Beijing Normal University, Beijing 100875, China

**Keywords:** freeze-thaw cycles, frozen duration, air temperature, snow water equivalent, soil moisture

## Abstract

The changes in near-surface soil freeze-thaw cycles (FTCs) are crucial to understanding the related hydrological and biological processes in terrestrial ecosystems under a changing climate. However, long-term dynamics of soil FTCs at the hemisphere scale and the underlying mechanisms are not well understood. In this study, the spatiotemporal patterns and main driving factors of soil FTCs across the Northern Hemisphere (NH) during 1979–2017 were analyzed using multisource data fusion and attribution approaches. Our results showed that the duration and the annual mean area of frozen soil in the NH decreased significantly at rates of 0.13 ± 0.04 days/year and 4.9 × 10^4^ km^2^/year, respectively, over the past 40 years. These were mainly because the date of frozen soil onset was significantly delayed by 0.1 ± 0.02 days/year, while the end of freezing and onset of thawing were substantially advanced by 0.21 ± 0.02 and 0.15 ± 0.03 days/year, respectively. Moreover, the interannual FTC changes were more drastic in Eurasia than in North America, especially at mid-latitudes (30°–45° N) and in Arctic regions (>75° N). More importantly, our results highlighted that near-surface air temperature (*T*_*a*_) and snowpack are the main driving factors of the spatiotemporal variations in soil FTCs. Furthermore, our results suggested that the long-term dynamics of soil FTCs at the hemisphere scale should be considered in terrestrial biosphere models to reduce uncertainties in future simulations.

## Public summary

• Patterns and driving factors of soil FTCs across the NH during 1979–2017 were analyzed
• The duration and annual mean area of frozen soil decreased significantly
• Interannual FTC changes were more drastic in Eurasia than in North America
• Near-surface air temperature and snow cover are the main factors in these variations

## Graphical abstract

**Figure:**
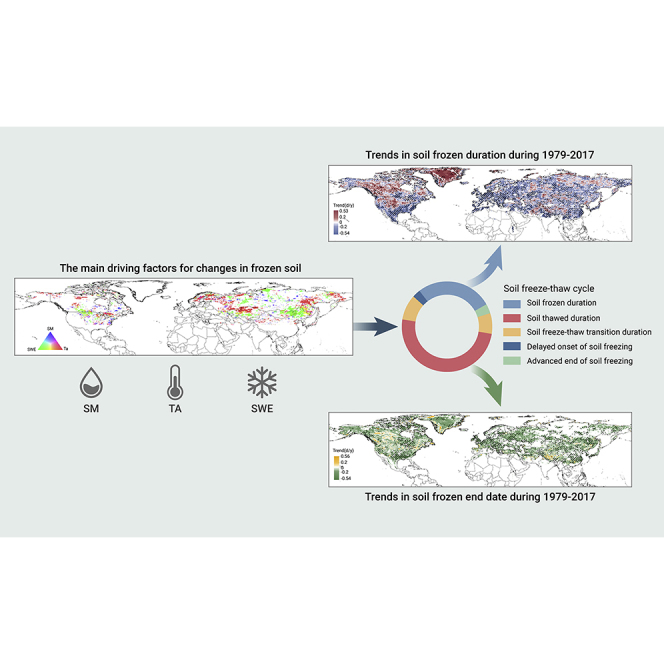

## Introduction

Near-surface soil freeze-thaw cycles (FTCs) are natural processes in which water in the soil freezes and thaws repeatedly due to periodic fluctuations in temperature at mid- to high latitudes as well as in high-altitude areas. Freeze-thaw processes lead to changes in physical and chemical properties and microbial activities by directly affecting the hydrothermal conditions of the soil,^1^ thereby influencing the land-atmosphere exchange of greenhouse gases.^2^ In addition to the long-term cumulative effects of FTCs on evapotranspiration and runoff dynamics,^3^ soil hydrothermal changes caused by abnormal FTCs on the Qinghai-Tibetan Plateau may have a marked impact on the East Asian atmospheric circulation, further altering the summer precipitation pattern in eastern China.^4^^,^^5^ In addition, the changes in plant ecophysiological processes induced by soil FTCs significantly influence vegetation productivity and structure in alpine ecosystems.^6^ In the context of global warming, the FTC pattern will likely experience more drastic variation and thus exert stronger impacts on terrestrial ecosystems, making FTC dynamics an important research topic.

It is estimated that seasonally frozen regions and intermittently frozen ground account for 50.6% and 6.6%, respectively, of the land area in the Northern Hemisphere (NH),^7^ indicating that more than half of the land in the NH experiences FTCs annually. Moreover, an obvious and rapid shift in soil FTC patterns has been recently observed.^8^ At high latitudes in particular, more frequent FTCs,^9^ an earlier thawing onset,^10^ a shallower freezing depth,^9^ and a reduced permafrost area^11^ have been reported. However, most previous studies have mainly focused on high-latitude regions in North America^12^ and Eurasia,^9^ and on the Qinghai-Tibetan Plateau,^13^ while relatively few investigations have been conducted at the hemisphere scale.^14^ In addition, the changes in the transitional state that occur between predominantly frozen and thawed states have rarely been investigated despite their ecological significance.^15^

Soil freeze-thaw processes are sensitive to the ambient environment, and the near-surface air temperature (*T*_*a*_), snow cover, and surface soil moisture (SM) have proven to be the main factors controlling changes in FTCs.^12^ Numerous investigations have found that an increasing *T*_*a*_ is responsible for reductions in soil freezing timing,^14^ duration,^16^ and depth.^17^ On the other hand, snow coverage and ablation processes can affect the land surface albedo, heat conductivity, and water mobility by insulating the soil from the atmosphere, thereby influencing FTC characteristics, including FTC frequency, intensity, and duration.^18^ However, the current literature mostly addresses the strong coupling between snow cover and FTCs from the perspective of snowpack parameters, such as its existence, extent, and duration, according to data retrieved by optical satellite remote sensing.^19^ With the development of detection techniques and algorithms, snow water equivalent (SWE) data retrieved from microwave sensors can provide information on the depth of snowpack, which may contribute to further illuminating the effects of snow properties on FTCs.^20^ SM is another main factor that controls FTCs. The initial soil water content determines freeze-thaw processes partly by affecting soil thermal conductivity.^21^ Despite a growing number of studies, large uncertainties still exist regarding the crucial drivers of FTC variation, especially in terms of the respective extents of the impacts of *T*_*a*_, SM, and snow cover.

Given the aforementioned issues, satellite-observed long-term consistent freeze-thaw state products and regression models were adopted in this study to obtain a comprehensive understanding of FTC variations across the NH. In addition, we performed multiple correlation analyses between the FTC dynamics and environmental factors. The objectives of this study were to (1) identify the spatiotemporal dynamics of soil FTC patterns (the duration, total area, and onset/end date of freezing and thawing states) across the NH during 1979–2017 and (2) evaluate the potential impacts and relative contributions of environmental factors to FTC variations.

## Results

### F/T duration changes

**Figure 1:**
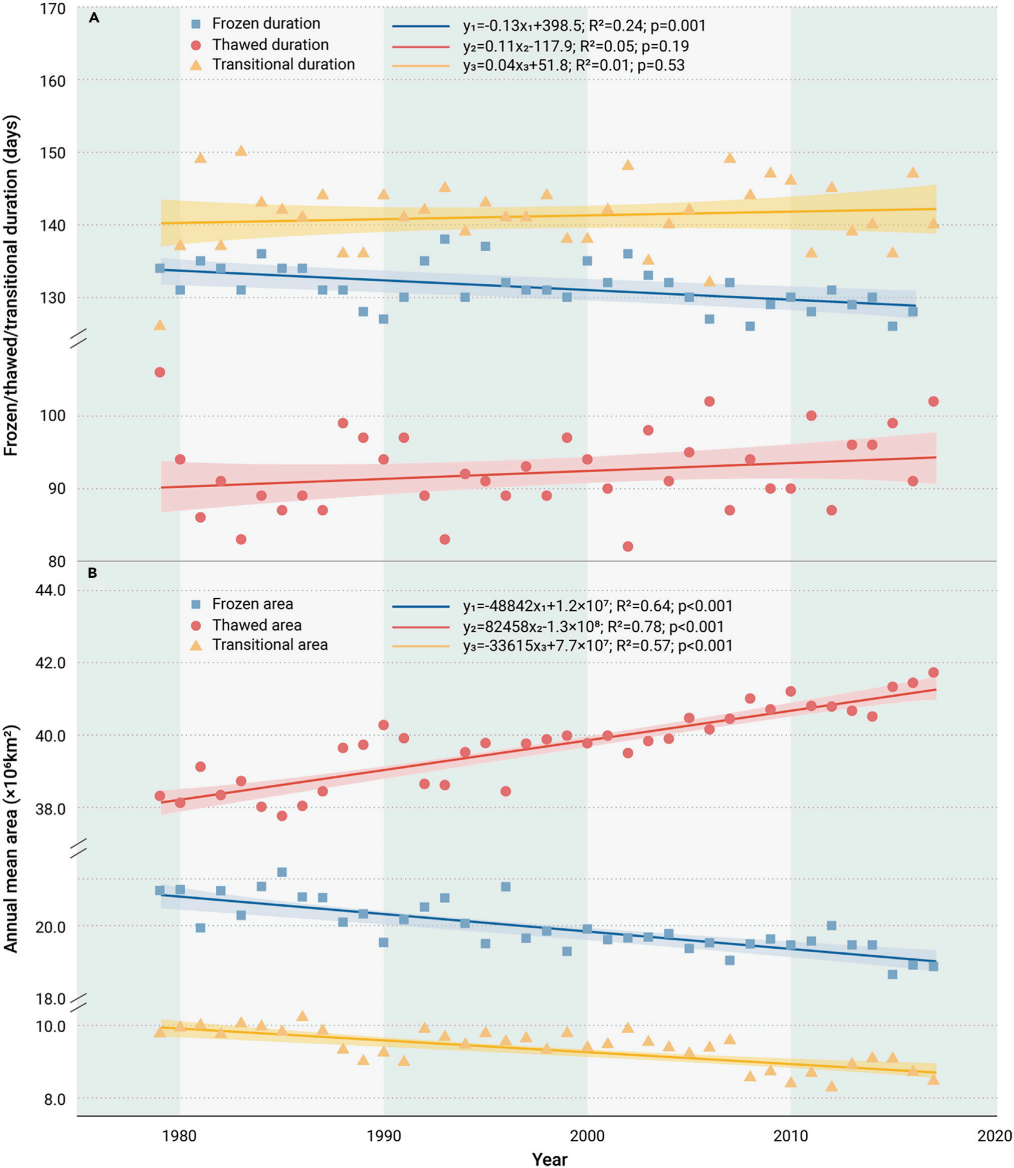
Chnges in the annual duration and the annual mean areas of different soil F/T states in the NH during 1979-2017 Interannual variations in the annual durations (A) and the annual mean areas (B) of the soil frozen/thawed/transitional state in the NH during 1979–2017. The shaded areas indicate the 95% confidence intervals.

The temporal changes in the F/T duration in the NH from 1979 to 2017 are shown in Figure 1A. The frozen duration was significantly shortened (p = 0.001) at a rate of 0.13 ± 0.04 days/year during the study period, while the increasing trends in the thawed duration (p = 0.19) and transitional duration (p = 0.53) were not significant, indicating that the frozen period turned into both thawed and transitional periods.

**Figure 2:**
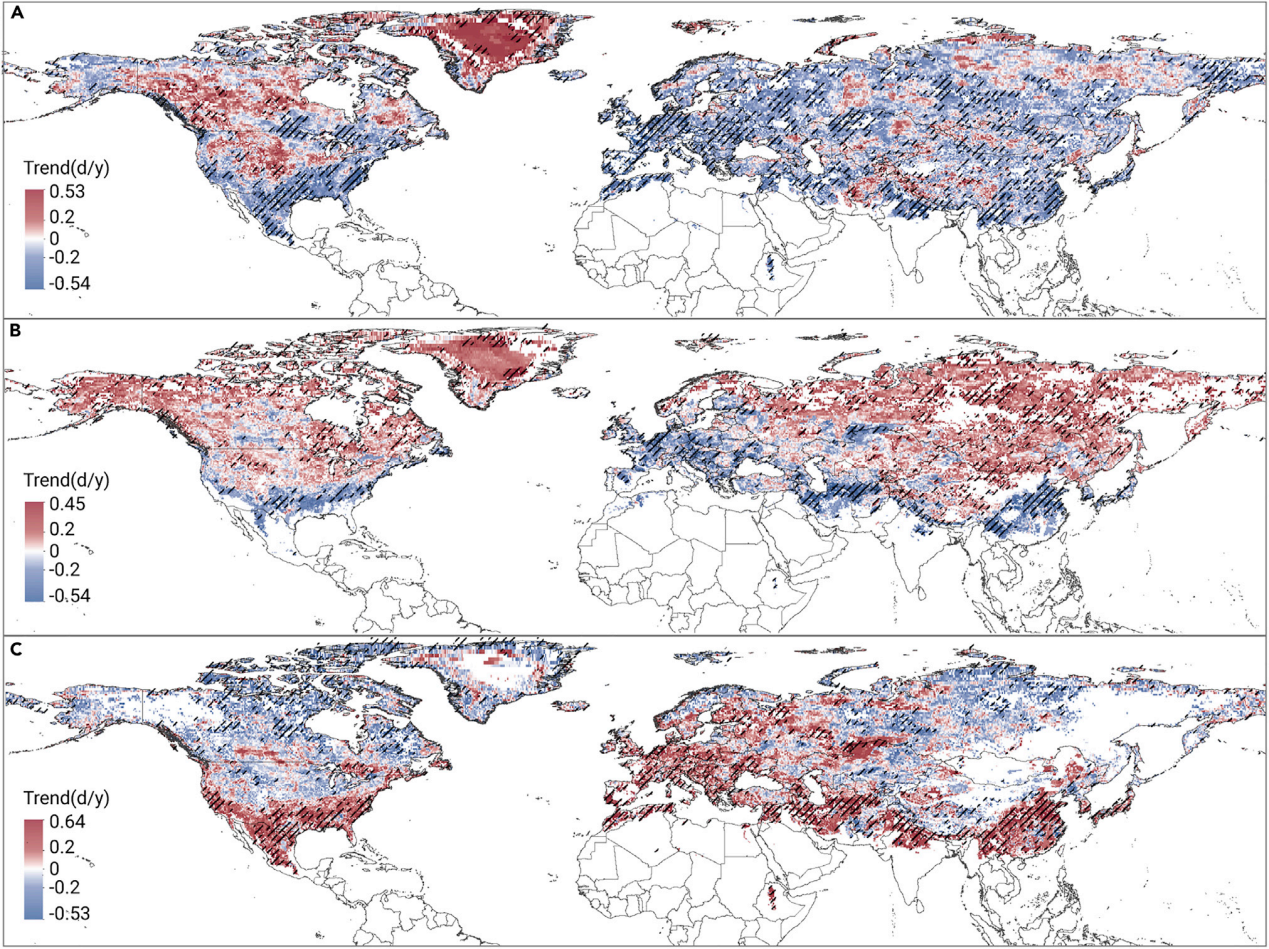
Spatial ditribution of changes in the annual duration of different soil F/T states in the NH during 1979-2017 Trends in the annual duration of soil frozen (A), thawed (B), and transitional (C) states during 1979–2017. Red and blue colors denote increases and decreases, respectively, with the shade of the colors indicating the magnitude of the trend. Areas with a significant change (p < 0.05) based on t test results are shaded.

The spatial patterns of the trends in the annual frozen, thawed, and transitional durations across the NH from 1979 to 2017 are illustrated in Figure 2. The interannual variations in the F/T duration in North America can be grouped into three types as follows: (1) in the Arctic region, in northwestern Canada, and on the Missouri Plateau in the United States, the frozen and thawed durations both increased, while the transitional duration declined sharply; (2) in Alaska, the frozen and transitional durations decreased, while the thawed duration increased; and (3) along the Pacific coastal mountains and the Appalachian Mountains, both the frozen and the thawed durations decreased due to the extension of the transitional period. On the other hand, in Eurasia, both the frozen and the transitional durations decreased with the increase in the thawed duration, except in Western Europe and on the Qinghai-Tibetan Plateau in Asia, where the frozen and thawed periods changed into transitional periods.

### F/T area changes

The annual mean frozen/thawed/transitional state area in the NH and their trends are shown in Figure 1B. The frozen area peaked at 2.15 × 10^7^ km^2^ (21.6% of the NH land area) in 1985 and was as small as 1.87 × 10^7^ km^2^ (18.8% of the NH land area) in 2015. The largest thawed area was 4.17 × 10^7^ km^2^ (42.0% of the NH land area) in 2017, and the smallest value was 3.78 × 10^7^ km^2^ (38.0% of the NH land area) in 1985. The transitional area was highest in 1986, with a value of 1.02 × 10^7^ km^2^ (10.3% of the NH land area), and was lowest in 2012, with a value of 8.28 × 10^6^ km^2^ (8.3% of the NH land area). The proportion of the annual mean thawed area in the NH was greater than the frozen and transitional state areas and is still expanding with the gradual shrinkage in the areas of the other two states. The annual mean frozen and transitional areas both decreased significantly (p < 0.001), at rates of 4.9 × 10^4^ and 3.4 × 10^4^ km^2^/year, respectively, while the thawed area increased by 8.2 × 10^4^ km^2^/year (p < 0.001).

**Figure 3:**
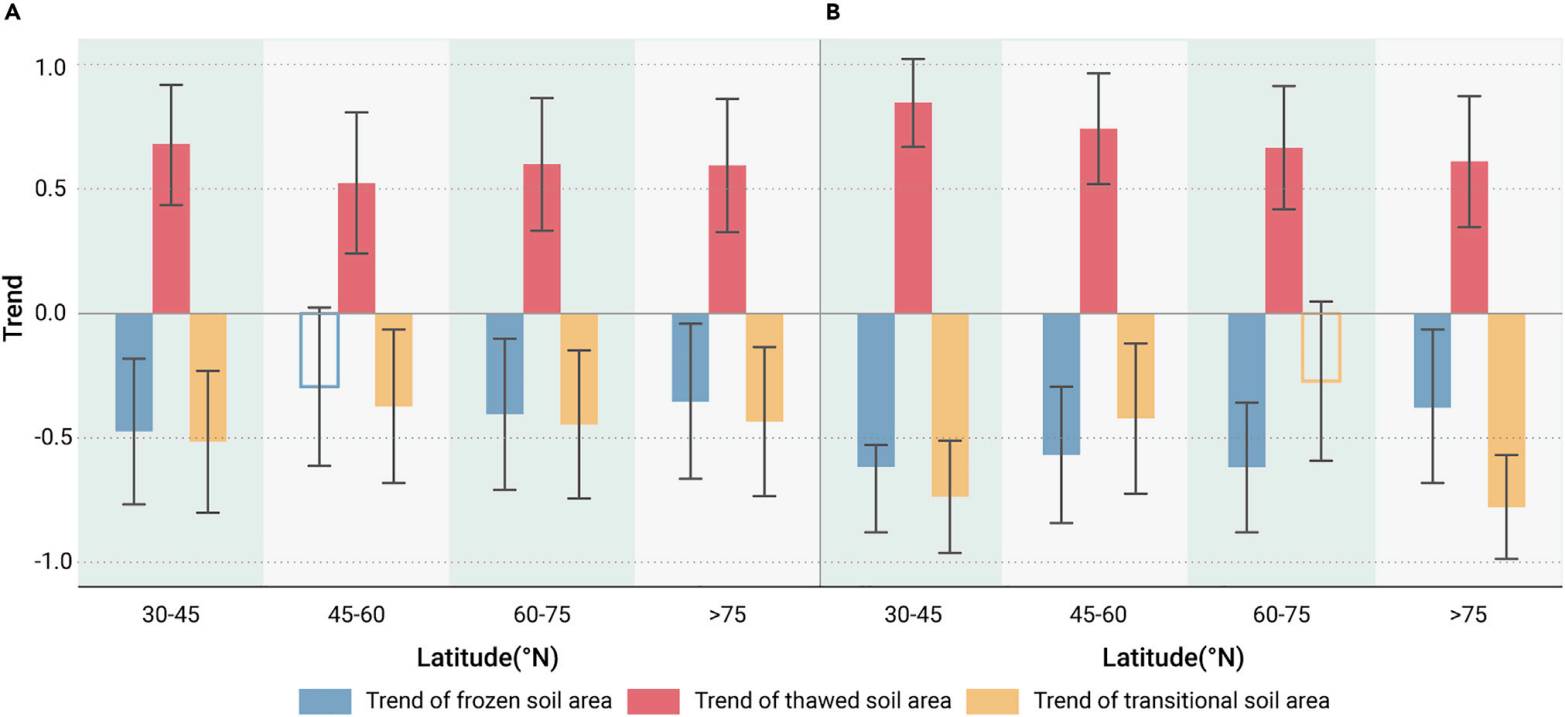
Changes in the annual mean areas of different soil F/T states across different latitudes in North America and Eurasia Trends in the annual mean areas (standardized by *Z* score) of soil frozen, thawed, and transitional states across different latitudes in North America (A) and Eurasia (B) (>30° N) from 1979 to 2017. Solid bars represent trends that are significant at the 95% significance level. The error bars represent the 95% confidence intervals of the regression coefficients (trends).

The trends in the annual mean F/T area varied with latitude in North America and Eurasia (Figure 3). Due to the small FTC-involved area at low latitudes (<30° N) and its drastic temporal variation under the distinct climate warming background, this part was excluded to avoid a misunderstanding of F/T annual area changes of the other latitudinal bands. In general, the expansion of the total thawed area and the shrinkage of the frozen and transitional areas were more drastic in Eurasia than in North America, especially at mid-latitudes (30°–45° N) and in Arctic regions (>75° N). However, at 45°–60° N in North America, the frozen soil area showed no significant trend, probably because a large mass of soil changed from the transitional state to the completely thawed state. Similarly, the nonsignificant change in the transitional area at 60°–75° N in Eurasia should be attributed to the direct transformation from frozen to thawed soil. North of 75° N in Eurasia, the sharp decrease in the transitional state area indicates a more severe freeze-thaw transition.

### Onset and end of soil F/T states

**Figure 4:**
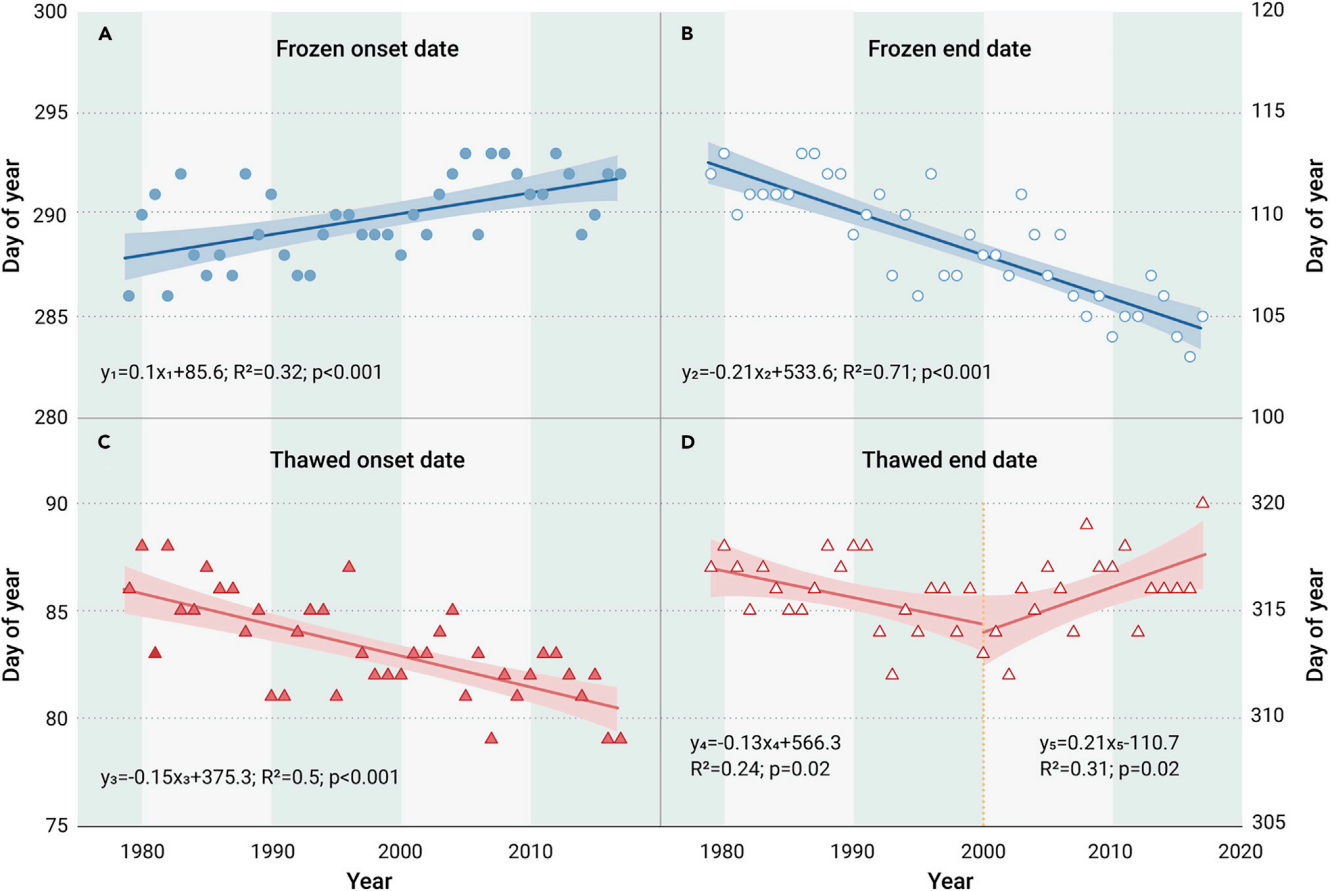
Changes in the onset and end of soil F/T states in the NH during 1979-2017 Temporal trends in the annual soil frozen onset (A), frozen end (B), thawed onset (C), and thawed end (D) dates in the NH from 1979 to 2017. Note that (A) minus (B) corresponds to the thawed duration, (D) plus (C) of the next year represents the frozen duration, (B) minus (C) corresponds to the spring transition duration, and (D) minus (A) represents the autumn transition duration. The shaded areas indicate the 95% confidence intervals.

Figure 4 illustrates the temporal trends in the end date of freezing/onset date of thawing in spring and the end date of thawing/onset date of freezing in autumn across the NH. For the freezing process, the onset date was significantly delayed at a rate of 0.10 ± 0.02 days/year (p < 0.001), and the end date was notably advanced by 0.21 ± 0.02 days/year (p < 0.001), which is consistent with the decreasing trend in the frozen duration (Figure 1A). The onset date of the thawed period came earlier, with a trend of 0.15 ± 0.03 days/year (p < 0.001), while the end date of the thawed period advanced at a rate of 0.13 ± 0.05 days/year (p = 0.02) before 2000, but was postponed by 0.21 ± 0.06 days/year (p = 0.04) later. The earlier dates of the onset of the thawed period and end of the frozen period indicate the overall advance of the spring transition (soil thawing takes place from early spring to summer in the NH). Although the fall transitional period (soil freezing occurs from early fall to winter in the NH) did not shorten from 1979 to 2000 due to the nonsignificant delay of the frozen onset date (p = 0.78), after 2000, the delays in both the date of the onset of freezing and the date of the end of the thawed period led to its overall postponement. The changes in the onset and end dates of soil thawing were consistent with the nonsignificant extension of the thawed and transitional period durations (Figure 1A).

**Figure 5:**
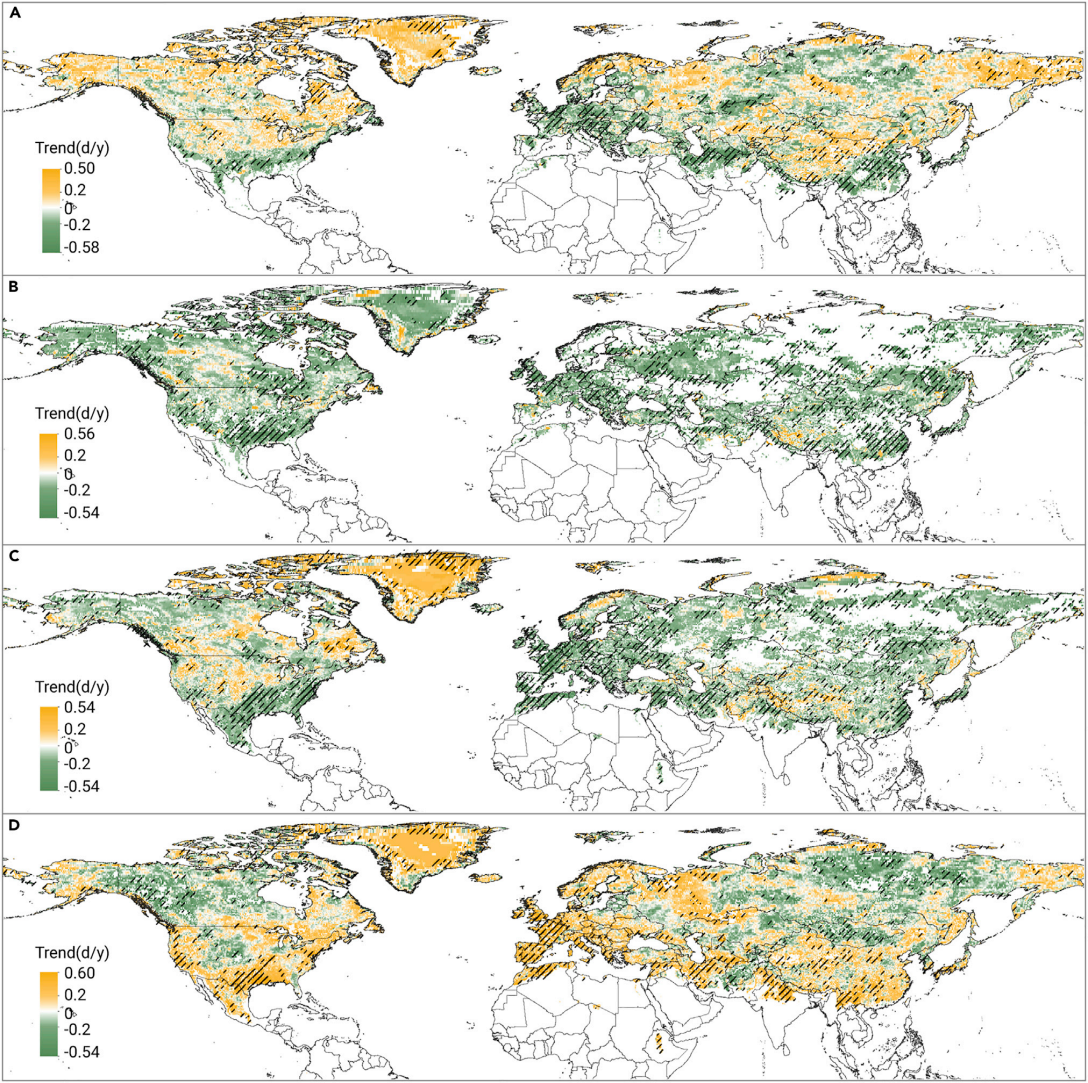
Spatial patterns of changes in the onset and end of soil F/T states in the NH during 1979-2017 Spatial patterns of the trends in the soil frozen onset (A), frozen end (B), thawed onset (C), and thawed end (D) dates in the NH from 1979 to 2017. Orange denotes a delay, green denotes an advance, and the shade of the colors indicates the magnitude of the trend. Areas with a significant change (p < 0.05) based on t test results are shaded.

The trends in the F/T onset and end dates showed high spatial variability as well (Figure 5). Despite the general pattern (i.e., delays in frozen onset and end of the thawed period, advanced onset of the thawed period and end of the frozen period), there were some exceptions in the Pacific coastal mountains, the Appalachian Mountains, and western Europe, where the frozen onset, frozen end, and thawed onset dates were all advanced, with the delay of the end of the thawed period only. This suggests that the transitional state extension in these areas (Figure 2C) was mainly due to the longer transitional period in autumn. In addition, in Arctic regions such as Greenland, the delay of thawed onset and the advance of the end of the frozen period mean that the frozen and thawed periods prolonged may be due to a shortened spring transitional period.

### Contributions of driving forces to the frozen duration

**Figure 6:**
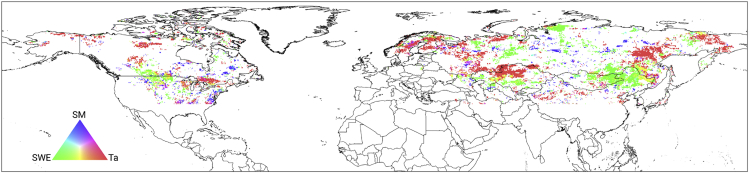
Geographic distribution of relative contributions of air temperature (*T*_*a*_), snow water equivalent (SWE), and soil moisture (SM) to the duration of frozen soil across the NH landmass (35°–85° N) from 1980 to 2017

The geographic distribution of the relative contributions of *T*_*a*_*,* SWE, and SM to the frozen duration in Figure 6 was obtained from the impact of single ambient driver factors (Figure S1). The *T*_*a*_-dominated regions were mainly distributed in northern and eastern Europe and northeastern Asia, while the SWE-dominated areas were mainly distributed across the Mongolian Plateau, eastern Europe, and mid-latitudes of North America. The influence of SM on frozen soil is not as extensive as those of *T*_*a*_ and SWE due to the sporadic distribution of the SM-dominated areas. In North America, the SM-dominated areas are distributed in the middle latitudes (south of 60° N), while in Eurasia, they are mainly distributed in the middle and high latitudes, such as Siberia (north of 60° N).

## Discussion

### Comparisons with previous studies

The results of this study indicated a general FTC change to a shortened frozen duration, a later onset of freezing, and an earlier end of freezing across the NH. Similar results have been found in China,^22^ the conterminous United States,^23^ and Mongolia,^24^ yet the magnitude of the change rates differed considerably among studies. For instance, based on ground meteorological data across China, Wang et al.^25^ found that the first date of freezing was delayed at a rate of 0.10 ± 0.03 days/year, the last date of freezing advanced at a rate of 0.15 ± 0.02 days/year, and the frozen duration decreased at a rate of 0.25 ± 0.04 days/year during 1956–2006. Another *in situ* observation result across China indicated that the first date of freezing was delayed by 0.20 ± 0.02 days/year, the last date of freezing was advanced by 0.36 ± 0.04 days/year, and the frozen duration was shortened by 0.56 ± 0.04 days/year during 1961–2011.^26^ These differences may be caused by variations in the research periods, analytical methods, and definitions of F/T states. In addition, the spatial distributions of transitional duration changes in this study are in line with previous results. The extension of transitional durations across the Pacific Coast Range and Appalachian Mountain regions of North America, western Europe, and the Qinghai-Tibetan Plateau was consistent with the results of previous satellite observation-based studies,^13^^,^^14^ whereas a shortening of transitional durations in Alaska and Greenland was also reported.^27^

### Effects of environmental factors on frozen soil

Changes in topsoil temperature are affected by near-surface *T*_*a*_ to a great extent, and this variable also affects the characteristics of the soil thermal state. Numerous observational data have demonstrated a warming hiatus of global surface air temperature from 1998 to 2012, which was induced by the El Niño/Southern Oscillation, solar irradiance fluctuations, and volcanic eruption.^28^ This phenomenon and its lagging effect may partly explain the delayed end date of soil thawing after 2000. In addition, the seasonal and diurnal asymmetry of the warming trend in the NH may influence the characteristics of FTCs. The published literature has demonstrated that the *T*_*a*_ in spring, summer, and autumn has increased by varying degrees,^29^^,^^30^ probably leading to changes in the end of freezing in spring and the onset date of freezing in fall. However, there remain controversies regarding whether winter is warming or cooling.^29^ For example, Jiménez-Muñoz indicated that the observed winter warming or cooling might depend on the temporal period considered and the methods used to calculate the trend and its significance.^31^ Subsequent studies reported nonsignificant winter warming at mid- and high latitudes in the NH,^32^ which was consistent with the geographical heterogeneity in frozen duration changes. Rapid daytime minimum temperatures rising in the Pacific coastal mountains, Appalachian Mountains, and western Asia were reported by Cox et al.,^33^ which was consistent with our results in Figure 5. These results indicate that the prolongation of the fall transitional period was likely due to the conversion from the complete freezing state to the transitional state of daytime thawing and night freezing in autumn.

SWE data retrieved by passive microwave sensors indicate the amount of accumulated snow on the land surface and the thickness of the snowpack.^34^ During the early stage of snow cover in autumn, the fresh and thin snowpack has a high albedo and weak insulating effect, resulting in cooling of the ground surface. With increasing thickness, the low heat conductivity of the stable snowpack decreases the correlation between the air and the soil temperatures and prevents soil freezing.^35^ On the other hand, a negative response of the frozen duration to SWE was observed in some areas (e.g., the Mongolian Plateau), where SWE is the dominant driving force (Figure 6), which is consistent with the results of previous investigations on the SWE trend (1980–2018)^36^ and snow cover duration trend (1982–2013)^37^ during almost the same period. Although the increased SWE and longer snow cover duration mean a later disappearance of snowpack, it does not mean a prolonged frozen soil duration. This is partly due to the upward transfer of heat flux from the deep soil as well as the fact that snowpack prevents heat from being released into the atmosphere. Both factors lead to an increase in the ground temperature and an earlier end to the frozen period than the onset of snowmelt.^35^ The relationship between the phenology of snow cover and soil freezing is complex, and further investigation is required.

Soil water directly participates in soil freeze-thaw processes, and it is an important factor in the soil FTC pattern. However, our results show that the areas mainly affected by SM were much smaller than those primarily influenced by *T*_*a*_ and snow cover. This might be mainly due to the fact that *T*_*a*_ and snow cover are the key factors controlling soil FTCs during winter periods in most regions, which could override the normal effect of SM. Moreover, previous studies have indicated that soil FTCs occur only when soil water content reaches a threshold value, which may vary with different soil properties, land covers, and climates.^2^^,^^21^ Therefore, another possible reason for the relatively small SM-dominated regions may be that the fluctuation range of SM in some regions did not exceed the threshold.

### Major uncertainties

Uncertainties may arise from the relatively coarse spatial resolution (∼25 km) of the FT-ESDR data. The mean brightness temperature retrievals in a grid cell might not reflect the difference in F/T states among different land cover types (vegetation, bare land, wetland, etc.). Compared with the Soil Moisture Active Passive and Advanced Microwave Scanning Radiometer 2 F/T records, the exclusive 37-GHz (vertical polarization) temperature brightness used to derive the FT-ESDR data is subject to the scattering effects of snow cover and other factors and has a shallower penetration depth.^38^ Thus, the retrieval accuracy of FT-ESDR is slightly lower than that of other F/T products in snow-covered areas.^39^ In addition, the reference threshold values used in MSTA were derived from the empirical relationship between the brightness temperature retrievals and the surface air temperature estimates. Shati et al.^40^ noted that the topsoil temperature (at 5 cm depth) may be more suitable than near-surface *T*_*a*_ (at 2 m height) for the detection of F/T states through intercomparison in high-latitude regions. Therefore, utilizing ground-based topsoil temperature data might be a potential way to improve the accuracy of F/T retrievals.

Wang et al.^41^ analyzed the contributions of SM, snow depth, and *T*_*a*_ to active layer thickness, and the results indicated that the Qinghai-Tibetan Plateau is a hotspot due to the significant contributions of *T*_*a*_ and SM. This result indicates that relative contribution assessments are deficient in these masked areas of SWE (south of 35° N, mountainous regions, and Greenland), which is another limitation in this study. In addition, topography and soil properties are relatively stable factors, and their impacts on FTC changes are not discussed in the present study. This assumption may affect the spatial pattern of the relative contributions of air temperature, SM, and snowpack.

### Conclusions

In this study, we investigated the spatiotemporal patterns and main driving factors of soil FTCs across the NH during 1979–2017. Our results clearly showed that the duration and the annual mean area of frozen soil decreased significantly due to freezing onset delay and freezing end advancement. The interannual FTC changes were more drastic in Eurasia than in North America, especially at mid-latitudes (30°–45° N) and in Arctic regions (>75° N). Moreover, our results demonstrated that *T*_*a*_ and snowpack rather than SM drove FTC pattern changes in the past 40 years. The variations in transitional and thawed soil states that occur during the warm season have a crucial role in vegetation growth and carbon sequestration, especially in high-latitude regions. Therefore, our results indicated that the hemispheric-scale spatiotemporal variations in soil FTCs are of prime importance to accurately predict the changes in hydrological and biological processes under future climate scenarios.

## Materials and methods

### Data source and processing

The up-to-date global long-term (1979–2017) microwave satellite Freeze/Thaw Earth System Date Record (FT-ESDR)^42^ (https://nsidc.org/data/nsidc-0477/versions/4) was used in this study. The frozen/thawed (F/T) classification in the dataset was achieved by comparing the microwave brightness temperature against a grid-specific threshold determined based on the empirical linear regression relationship between brightness temperature and surface air temperature in that grid. This algorithm, called the modified seasonal threshold algorithm (MSTA), can promote consistency among the F/T records derived from observations by different microwave sensors (the Scanning Multichannel Microwave Radiometer [SMMR], the Special Sensor Microwave/Imager [SSM/I], and the Special Sensor Microwave Imager/Sounder [SSMIS]) over 40 years. Compared with the *in situ* surface air temperatures, the mean annual spatial classification accuracies were approximately 90.3% and 84.3% for evening (a.m.) and morning (p.m.) overpass retrievals, respectively.^42^ The FT-ESDR was reprojected from the Equal-Area Scalable Earth Grid projection with ∼25 km grid resolution to the WGS1984 geographic coordinate system with a resolution of 0.25°.

Monthly *T*_*a*_ records at 2 m from 1979 to 2017 were acquired from ERA5, the fifth-generation European Centre for Medium-Range Weather Forecasts atmospheric reanalysis of the global climate (https://doi.org/10.24381/cds.adbb2d47). This dataset has been greatly improved compared with its predecessor, ERA-Interim, due to the use of a significantly more advanced 4D-Var assimilation scheme.

SWE data were acquired from the latest GlobSnow v.3.0 SWE records developed by the European Space Agency (https://www.globsnow.info/). By combining the satellite data retrieved from the SMMR, SSM/I, and SSMIS sensors and meteorological station-based climatic data, GlobSnow v.3.0 provides monthly SWE data (35°–85° N of the NH land surface area, excluding the alpine areas and Greenland) from 1979 to 2018. Based on Bayesian statistical inversion theory and the Helsinki University of Technology snow emission model, GlobSnow v.3.0 has been shown to have a higher consistency than other hemisphere-scale SWE data. This dataset, with ∼25 km resolution in Lambert's equal-area azimuthal projection, has also been converted to WGS1984 with a resolution of 0.25°.

Surface SM data were derived from the Global Land Evaporation Amsterdam Model (GLEAM) v.3.5a (www.gleam.eu), a global dataset that was produced using satellite-observed SM, vegetation optical depth and SWE, reanalysis air temperature and radiation, and a multisource-based precipitation product. This dataset spans a 41-year period from 1980 to 2020 and is provided on a 0.25° spatial resolution with a daily temporal resolution. Validating against 2,325 *in situ* SM measurements and 91 eddy-covariance towers, the correlation of surface SM increases from 0.61 to 0.64 compared with the previous version of GLEAM (v.2).^43^ Further information regarding the datasets can be found in the supplemental material.

### Analytical methods

In this study, a year was divided into three parts according to the daily F/T state: frozen, thawed, and transitional periods. Based on the a.m. and p.m. F/T states derived from the ascending and descending orbits, respectively, the daily F/T states were classified into four categories: frozen (a.m. frozen, p.m. frozen), thawed (a.m. thawed, p.m. thawed), transitional (a.m. frozen, p.m. thawed), and inverse transitional (a.m. thawed, p.m. frozen). Because the occurrence of the inverse transitional state is mainly attributed to the overpassing time of the sensors and the determination algorithm of the MSTA threshold,^44^ while only a small area is involved, the inverse transitional state was also classified as a transitional period.

#### Trend analysis

To conduct a consistent long-term analysis, July 1 to June 30 of the following year was defined as an analysis year. Accordingly, the onset and end dates of surface soil freezing were defined as the first day after July 1 and the last day before June 30 in the next year, respectively, when a grid had been frozen for three successive days, while the onset and end dates of soil thawing were similarly defined. Therefore, the completely frozen (thawed) duration of near-surface soil can be calculated as the number of days between the end and the onset of soil thawing (freezing), while the duration of the transitional state can be calculated according to Equation (1):

(Equation 1) $$D_{tran}=\;F_{off}-\;T_{on\;}+\;T_{off}-F_{on},$$

where D_tran_ is the duration of the transitional state, F_on_ and F_off_ are the onset and end dates of the freezing state in a year, respectively, and T_on_ and T_off_ are the onset and end dates of the thawing state, respectively.^22^

After calculating the annual durations of the frozen, thawed, and transitional states by pixel, we used the Mann-Kendall (M-K) trend test to identify the potential monotonic increasing/decreasing trend. For pixels passing the M-K significance test at the 95% significance level, linear regression slopes were calculated to derive the spatial map of the F/T duration trend in the NH from 1979 to 2017. Next, using the same method, we calculated the temporal trends in the annual mean F/T area in the NH as well as at different latitudes in North America and Eurasia after quantifying the daily total frozen/thawed/transitional area in the NH and calculating the annual average.

#### Piecewise linear regression

A piecewise linear regression model that is continuous at the turning point was constructed to detect potential changes in the F/T duration, annual area, and onset and end date trends (Figures 1 and 4). We searched for the potential turning point based on the criterion of the least residual sum of squares. However, if the difference between the slopes before and after that point was nonsignificant (p > 0.05 or the 95% confidence intervals of the two slopes overlapped) or if neither of the trends before and after the turning point was significant, we determined that there was no turning point throughout the entire period.^45^

#### Contributions of environmental drivers

We assessed multicollinearity between *T*_*a*_, SM, and SWE using the variance inflation factor, and the results indicated no obvious collinearity between them. To eliminate the covariate effects between *T*_*a*_, SWE, and SM, we conducted pixel-level partial correlation analysis between the annual frozen duration and the mean *T*_*a*_/SWE/SM from 1979 to 2017, which helps to reveal the contributions of various environmental factors to soil FTCs (Figure 6). The statistical significance of the partial correlation at the 95% confidence level was determined based on the two-tailed Student t test. All the variables were normalized and standardized using *Z*-score transformation before the correlation analyses.

All statistical analyses described above were conducted using MATLAB R2014b (MathWorks, Natick, MA). Figures were created using ArcGIS 10.5 software (http://www.esri.com/) and Origin 2018 software (OriginLab Corporation, Northampton, MA).

## Lead contact website

Bo-Jie Fu: http://dse.rcees.cas.cn/yjzjs/qyjggjystgc/jggjystgcyjz/201211/t20121116_101976.html.

Xing Wu: http://dse.rcees.cas.cn/yjzjs/qyjggjystgc/jggjystgcyjz/201211/t20121119_101998.html.

## Supplemental information

Document S1. Supplemental methods and Figure S1

Document S2. Article plus supplemental information

## References

[1] Guo D., Yang M., Wang H. (2011). Characteristics of land surface heat and water exchange under different soil freeze/thaw conditions over the central Tibetan Plateau. Hydrol. Process..

[2] Wu X., Brueggemann N., Gasche R. (2010). Environmental controls over soil-atmosphere exchange of N_2_O, NO, and CO_2_ in a temperate Norway spruce forest. Glob. Biogeochem Cycles.

[3] Zheng D.H., van der Velde R., Su Z.B. (2018). Impact of soil freeze-thaw mechanism on the runoff dynamics of two Tibetan rivers. J. Hydrol..

[4] Wang C., Zhao W., Cui Y. (2020). Changes in the seasonally frozen ground over the eastern Qinghai-Tibet Plateau in the past 60 years. Front Earth Sci..

[5] Wei K., Ouyang C., Duan H. (2020). Reflections on the catastrophic 2020 Yangtze river basin flooding in southern China. The Innovation.

[6] Kim Y., Kimball J.S., Didan K. (2014). Response of vegetation growth and productivity to spring climate indicators in the conterminous United States derived from satellite remote sensing data fusion. Agr For. Meteorol.

[7] Zhang, T., Barry, R., Knowles, K., et al. (2003). DiStribution of seasonally and perennially frozen ground in the Northern Hemisphere. Paper presented at: Proceedings of the 8th International Conference on Permafrost (AA Balkema Publishers Zürich, Switzerland).

[8] Hock R., Rasul G., Adler C. (2019). IPCC Special Report on the Ocean and Cryosphere in a Changing Climate.

[9] Frauenfeld O.W., Zhang T. (2011). An observational 71-year history of seasonally frozen ground changes in the Eurasian high latitudes. Environ. Res. Lett..

[10] Schwartz M.D., Ahas R., Aasa A. (2006). Onset of spring starting earlier across the Northern Hemisphere. Glob. Change Biol.

[11] Shi Y.Y., Niu F.J., Lin Z.J. (2019). Freezing/thawing index variations over the circum-Arctic from 1901 to 2015 and the permafrost extent. Sci. Total Environ..

[12] Henry H.A.L. (2008). Climate change and soil freezing dynamics: historical trends and projected changes. Clim. Change.

[13] Li X., Jin R., Pan X. (2012). Changes in the near-surface soil freeze-thaw cycle on the Qinghai-Tibetan Plateau. Int. J. Appl. Earth Obs Geoinf.

[14] Smith N.V., Saatchi S.S., Randerson J.T. (2004). Trends in high northern latitude soil freeze and thaw cycles from 1988 to 2002. J. Geophys. Res-atmos..

[15] Moser J.G., Oberbauer S.F., Sternberg L.D.S.L. (2016). Water uptake of Alaskan tundra evergreens during the winter-spring transition. Am. J. Bot..

[16] Wang K., Zhang T., Guo H. (2016). Climatology of the timing and duration of the near-surface soil freeze-thaw status across China. Arct Antarct Alp Res..

[17] Peng X., Zhang T., Frauenfeld O.W. (2017). Response of seasonal soil freeze depth to climate change across China. Cryosphere.

[18] Fu Q., Hou R., Li T. (2018). The functions of soil water and heat transfer to the environment and associated response mechanisms under different snow cover conditions. Geoderma.

[19] Kim Y., Kimball J.S., Robinson D.A. (2015). New satellite climate data records indicate strong coupling between recent frozen season changes and snow cover over high northern latitudes. Environ. Res. Lett..

[20] Yi Y., Kimball J.S., Chen R.H. (2019). Sensitivity of active-layer freezing process to snow cover in Arctic Alaska. Cryosphere.

[21] Sun L., Chang X., Yu X. (2021). Effect of freeze-thaw processes on soil water transport of farmland in a semi-arid area. Agric. Water Manage.

[22] Han L., Tsunekawa A., Tsubo M. (2010). Monitoring near-surface soil freeze-thaw cycles in northern China and Mongolia from 1998 to 2007. Int. J. Appl. Earth Obs Geoinf.

[23] McCabe G.J., Betancourt J.L., Feng S. (2015). Variability in the start, end, and length of frost-free periods across the conterminous United States during the past century. Int. J. Climatol.

[24] Wu T., Wang Q., Zhao L. (2011). Observed trends in surface freezing/thawing index over the period 1987–2005 in Mongolia. Cold Regions Sci. Tech..

[25] Wang K., Zhang T., Zhong X. (2015). Changes in the timing and duration of the near-surface soil freeze/thaw status from 1956 to 2006 across China. Cryosphere.

[26] Wang X.Q., Chen R.S., Liu G.H. (2019). Spatial distributions and temporal variations of the near-surface soil freeze state across China under climate change. Glob. Planet Change.

[27] Barichivich J., Briffa K.R., Myneni R.B. (2013). Large-scale variations in the vegetation growing season and annual cycle of atmospheric CO_2_ at high northern latitudes from 1950 to 2011. Glob. Change Biol.

[28] Trenberth K.E., Fasullo J.T. (2013). An apparent hiatus in global warming?. Earth’s Future.

[29] Cohen J.L., Furtado J.C., Barlow M. (2012). Asymmetric seasonal temperature trends. Geophys. Res. Lett..

[30] Cheng H. (2020). Future earth and sustainable developments. The Innovation.

[31] Jimenez-Munoz J.C., Sobrino J.A., Mattar C. (2013). Has the Northern Hemisphere been warming or cooling during the boreal winter of the last few decades?. Glob. Planet Change.

[32] Ji S.N., Classen A.T., Zhang Z.H. (2017). Asymmetric winter warming advanced plant phenology to a greater extent than symmetric warming in an alpine meadow. Funct. Ecol..

[33] Cox D.T.C., Maclean I.M.D., Gardner A.S. (2020). Global variation in diurnal asymmetry in temperature, cloud cover, specific humidity and precipitation and its association with leaf area index. Glob. Change Biol.

[34] Saberi N., Kelly R., Flemming M. (2019). Review of snow water equivalent retrieval methods using spaceborne passive microwave radiometry. Int. J. Remote Sens.

[35] Zhang T.J. (2005). Influence of the seasonal snow cover on the ground thermal regime: an overview. Rev. Geophys..

[36] Pulliainen J., Luojus K., Derksen C. (2020). Patterns and trends of Northern Hemisphere snow mass from 1980 to 2018. Nature.

[37] Chen X., Liang S., Cao Y. (2016). Satellite observed changes in the Northern Hemisphere snow cover phenology and the associated radiative forcing and feedback between 1982 and 2013. Environ. Res. Lett..

[38] Wang J., Jiang L., Cui H. (2020). Evaluation and analysis of SMAP, AMSR2 and MEaSUREs freeze/thaw products in China. Remote Sens Environ..

[39] Prince M., Roy A., Brucker L. (2018). Northern Hemisphere surface freeze-thaw product from Aquarius L-band radiometers. Earth Syst. Sci. Data.

[40] Shati F., Prakash S., Norouzi H. (2018). Assessment of differences between near-surface air and soil temperatures for reliable detection of high-latitude freeze and thaw states. Cold Regions Sci. Tech..

[41] Wang C., Wu D., Kong Y. (2017). Changes of soil thermal and hydraulic regimes in northern hemisphere permafrost regions over the 21st century. Arct Antarct Alp Res..

[42] Kim Y., Kimball J.S., Glassy J. (2017). An extended global Earth system data record on daily landscape freeze-thaw status determined from satellite passive microwave remote sensing. Earth Syst. Sci. Data.

[43] Martens B., Miralles D.G., Lievens H. (2017). GLEAM v3: satellite-based land evaporation and root-zone soil moisture. Geoscientific Model Development.

[44] Kim Y., Kimball J.S., McDonald K.C. (2011). Developing a global data record of daily landscape freeze/thaw status using satellite passive microwave remote sensing. IEEE Trans. Geosci. Remote Sens.

[45] Pan N., Feng X., Fu B. (2018). Increasing global vegetation browning hidden in overall vegetation greening: Insights from time-varying trends. Remote Sens Environ..

