# Supplementary material for: Shortened duration and reduced area of frozen soil in the Northern Hemisphere
Source: Innovation (Camb). 2021 Jul 21;2(3):100146. doi: 10.1016/j.xinn.2021.100146 (PMC8454614; doi:10.1016/j.xinn.2021.100146)
Supplement: Document S1. Supplemental methods and Figure S1 [file mmc1.pdf]

The Innovation, Volume *2*

Supplemental Information

Shortened duration and reduced area of frozen soil in the Northern Hemisphere

Ting Li, Yong-Zhe Chen, Li-Jian Han, Lin-Hai Cheng, Yi-He Lv, Bo-Jie Fu, Xiao-Ming Feng, and Xing Wu

Supplemental Information

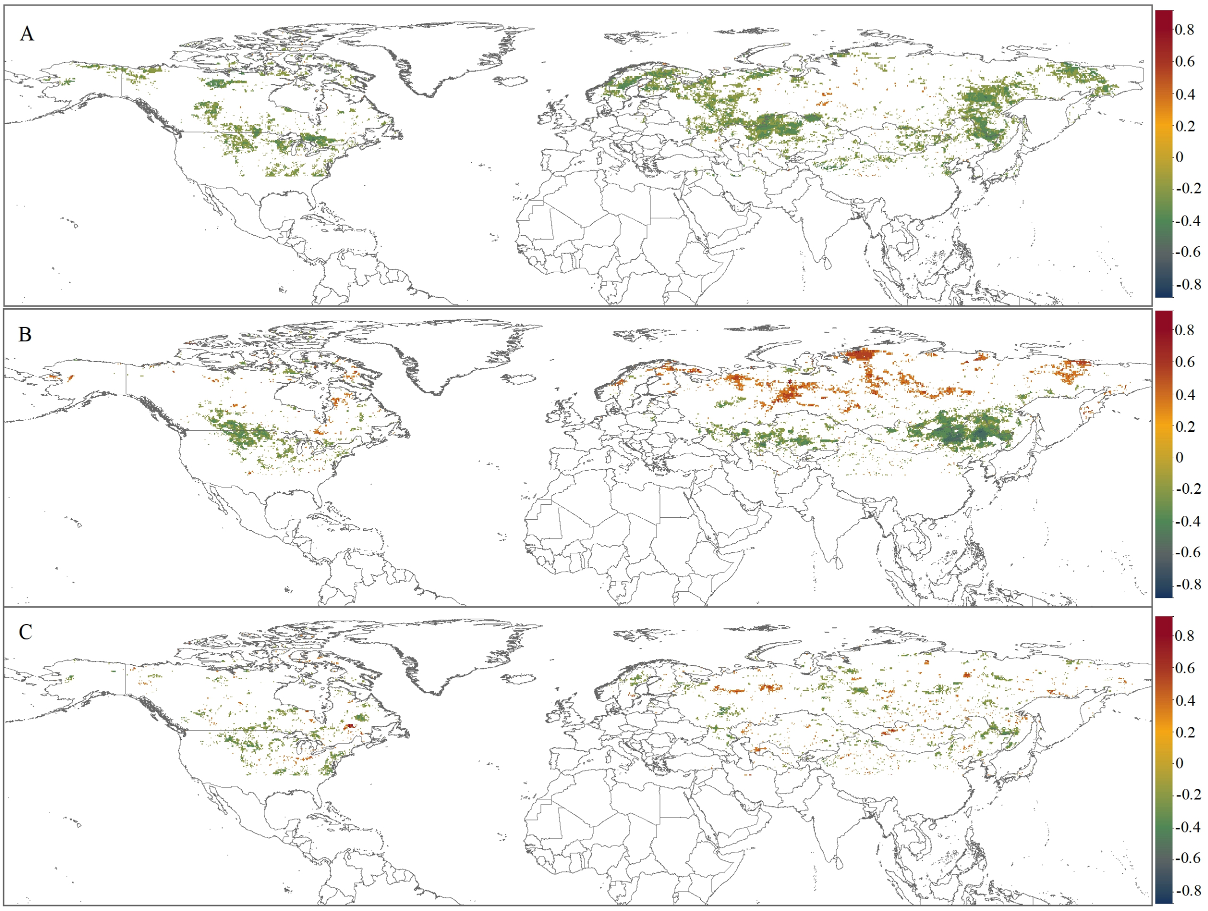

Figure S1. Spatial distribution of the partial correlation coefficients (statistically significant with $p<0.05$) between the duration of frozen soil during 1979-2017 and T_a (A)/SWE (B)/SM (C) across the NH landmass (35 to 85°N). Colorbars represent the partial correlation coefficient values.

Further information regarding the datasets

1. The satellite soil freeze/thaw Earth system dataset (FT-ESDR)

The FT-ESDR was generated by calibrating 37GHz brightness temperature retrievals from the Scanning Multichannel Microwave Radiometer (SMMR), Special Sensor Microwave Imager (SSM/I), and SSM/I Sounder (SSMIS). The modified seasonal threshold algorithm was applied to define the freeze/thaw (F/T) status. More details can find in Kim *et al.*[1] and the data user guide (https://nsidc.org/data/nsidc-0477).

The F/T ESDR accuracy shows strong seasonal and annual variability and is reduced during active F/T transition periods when spatial heterogeneity in landscape F/T processes is maximized in relation to the relatively coarse (~25 km) satellite footprint.[2] The accuracy against ground observation was evaluated through grid-cell-to-point comparisons with WMO surface air temperature (SAT). The mean annual FT spatial classification accuracy relative to WMO weather station SAT measurements was approximately 90.3% (PM overpass) and 84.3% (AM over- pass) over the global FT-ESDR domain during 1979 to 2014.[1]

2. ERA5 air temperature dataset

ERA5 combines vast amounts of historical observations into global estimates using advanced modelling and data assimilation systems (https://rmets.onlinelibrary.wiley.com/doi/10.1002/qj.3803). Based on the Integrated Forecasting System (IFS) Cy41r2 which was operational in 2016, ERA5 thus benefits from a decade of developments in model physics, core dynamics and data assimilation. Since April 2019, ERA5 analyses of 2m temperature are used as input to monthly summaries published by the C3S. Before that they had been based on ERA-Interim for almost 4 years. Such analyses from ERA-Interim, and earlier ones from ERA-40, have been shown to be of reasonable quality and complementary to the products of conventional analyses of climatological station data.

The trend and low frequency variability of global-mean temperature from ERA5 are largely consistent with values provided by other datasets from 1950 onwards. ERA5 is biased cold over the majority of the land surface prior to 1967 because the cold bias of its background forecasts is less well constrained by analyzed observations then than in later years. The effect is smaller, however, than the uncertainty in all datasets that arises from differences in sea-surface temperature (SST) analysis, and no larger than is estimated to arise in some datasets from using SST rather than air temperature over sea. ERA5's use of marine air temperature rather than SST is also one reason its global temperatures are a little higher than those from other datasets for the latest few years.

ERA5 performs relatively well for Europe from 1950 onwards, but uncertainty is somewhat larger in the mid-1960s, when there are significant gaps in observational data coverage. Its worst persistent temperature bias is over Australia prior to the 1970s. The issue in this case is not only lack of surface observations but also an unusually large warm bias of the background forecasts. Aside from this, the ERA5 surface analysis scheme does not cope well with the preponderance of observations from Australia that are for non-standard times. In addition, agreement over Australia between several reanalyses and monthly climatological datasets tends to be poorer during occasional wet spells. This stems in part at least from different definitions of daily average temperature. A number of issues elsewhere, of a more-local nature, have been identified. They relate to data gaps, questionable representation of fractional sea-ice cover, inconsistent coastal SSTs and erroneous temperatures of the Great Lakes(https://confluence.ecmwf.int/display/CKB/ERA5%3A+data+documentation#ERA5:datadocumentation-Observations) (https://www.ecmwf.int/en/elibrary/19911-low-frequency-variability-and-trends-surface-air-temperature-and-humidity-era5-and).

3. GlobSnow v3.0 snow water equivalent (SWE) data

The record on snow water equivalent (SWE) is produced using a combination of passive microwave radiometer and ground-based weather station data, spanning years

1979 to 2018 in EASE-Grid projection with a nominal spatial resolution of 25 km. GlobSnow v3.0 SWE Dataset constructed by combining satellite-based passive microwave radiometer data (Nimbus-7 SMMR, DMSP 5D2 SSM/I and DMSP 5D3 SSMIS) with ground based synoptic snow depth observations using bayesian data assimilation, incorporating HUT Snow Emission model; by Pulliainen et al.[3] and Lemmetyinen et al.[4]

The potential uncertainties was investigated by Venäläinen *et al.*[5] Compared with earlier GlobSnow versions 2, major changes have been made for v3.0, the version used here. These changes include spatiotemporal homogenization of applied synoptic weather-station snow depth observations, consideration of lake ice in the forward model of spaceborne-observed microwave brightness temperature, and revised modelling of forest canopy brightness temperature and microwave attenuation in the forest canopy. In order to constrain the number of variables in the SWE retrieval procedure, the snowpack is considered as a single layer with a constant density (0.24 g/cm^3).[5]

4. GLEAM v3.5 surface soil moisture (SM) dataset

The Global Land Evaporation Amsterdam Model (GLEAM) is a set of algorithms that separately estimate the different components of land evaporation: transpiration, bare-soil evaporation, interception loss, open-water evaporation and sublimation. Additionally, GLEAM provides surface and root-zone soil moisture, potential evaporation and evaporative stress conditions (**https://www.gleam.eu/**). This dataset spans a 41-year period from 1980-2020 and is provided on a 0.25 degree spatial resolution with a daily temporal resolution.

Validating against an extensive set of in situ measured evaporation and soil moisture, the v3a soil moisture data set shows a slightly higher quality as compared to the other two data sets. The higher accuracy of the v3a soil moisture is explained by the high quality of the MSWEP precipitation forcing over the regions where soil moisture probes are located, compared to the satellite-based forcing in the v3b and v3c data sets. Results, however, might be biased given that the vast majority (i.e. more

than 75 %) of the in situ soil moisture sites are located in the CONUS, where gauge-based precipitation products are known to outperform satellite products. Finally, the quality of the new v3 data sets is also compared to analogous data sets obtained using GLEAM v2. For the soil moisture, the modifications in GLEAM result in a consistent improvement across the vertical profile. These improvements mainly relate to the optimized drainage algorithm and the new data assimilation system, which allow a more realistic representation of the downward flux of water through the soil profile.[6]

References

1. Kim, Y., Kimball, J.S., Glassy, J., et al. (2017). An extended global Earth system data record on daily landscape freeze-thaw status determined from satellite passive microwave remote sensing. Earth Syst Sci Data, ***9***, 133-147.
2. Kim, Y., Kimball, J.S., McDonald, K.C., et al. (2011). Developing a Global Data Record of Daily Landscape Freeze/Thaw Status Using Satellite Passive Microwave Remote Sensing. IEEE Trans Geosci Remote Sens, ***49***, 949-960.
3. Pulliainen, J.T., Grandell, J., and Hallikainen, M.T. (1999). HUT snow emission model and its applicability to snow water equivalent retrieval. IEEE Trans Geosci Remote Sens, ***37***, 1378-1390.
4. Lemmetyinen, J., Pulliainen, J., Rees, A., et al. (2010). Multiple-layer adaptation of HUT snow emission model: comparison with experimental data. IEEE Trans Geosci Remote Sens, ***48***, 2781-2794.
5. Venäläinen, P., Luojus, K., Lemmetyinen, J., et al. (2021). Impact of dynamic snow density on GlobSnow snow water equivalent retrieval accuracy. The Cryosphere, ***15***, 2969-2981.
6. Martens, B., Miralles, D.G., Lievens, H., et al. (2017). GLEAM v3: satellite-based land evaporation and root-zone soil moisture. Geoscientific Model Development, ***10***, 1903-1925.
